# Recurrence of tuberculosis among newly diagnosed sputum positive pulmonary tuberculosis patients treated under the Revised National Tuberculosis Control Programme, India: A multi-centric prospective study

**DOI:** 10.1371/journal.pone.0200150

**Published:** 2018-07-06

**Authors:** Banurekha Velayutham, Vineet Kumar Chadha, Neeta Singla, Pratibha Narang, Vikas Gangadhar Rao, Sanjeev Nair, Srinivasan Ramalingam, Gomathi Narayanan Sivaramakrishnan, Bency Joseph, Sriram Selvaraju, Shivakumar Shanmugam, Rahul Narang, Praseeja Pachikkaran, Jyothi Bhat, Chinnaiyan Ponnuraja, Bhoomika Bajaj Bhalla, Bhadravathi Amarnath Shivashankara, George Sebastian, Rajiv Yadav, Ravendra Kumar Sharma, Rohit Sarin, Vithal Prasad Myneedu, Rupak Singla, Khalidumer Khayyam, Sunil Kumar Mrithunjayan, Subramonia Pillai Jayasankar, Praveen Sanker, Krishnaveni Viswanathan, Rajeevan Viswambharan, Kapil Mathuria, Manpreet Bhalla, Nitu Singh, Kondeshvar Balaji Tumane, Ajay Dawale, Chandra Prakash Tiwari, Radhelal Bansod, Lavanya Jayabal, Lakshmi Murali, Sunil D. Khaparde, Raghuram Rao, Mohideen S. Jawahar, Mohan Natrajan

**Affiliations:** 1 ICMR-National Institute for Research in Tuberculosis (NIRT), Chennai, India; 2 National Tuberculosis Institute (NTI), Bangalore, Karnataka, India; 3 National Institute of Tuberculosis and Respiratory Diseases (NITRD), New Delhi, India; 4 Mahatma Gandhi Institute of Medical Sciences (MGIMS), Sevagram, Wardha, Maharashtra, India; 5 ICMR-National Institute for Research in Tribal Health (NIRTH), Jabalpur, Madhya Pradesh, India; 6 Thiruvananthapuram Medical College, Thiruvananthapuram (TMCT), Kerala, India; 7 State TB Centre, Kerala, India; 8 Directorate of Health Services, Thiruvananthapuram, Kerala, India; 9 District TB Centre, Kollam, Kerala, India; 10 District TB Centre, Malviya Nagar, India; 11 District TB Centre, Nagpur, India; 12 District TB Centre, Wardha, India; 13 District TB Centre, Jabalpur, India; 14 District TB Centre, Mandla, India; 15 District TB Centre, Chennai, India; 16 District TB Centre, Thiruvallur, India; 17 Central TB Division, New Delhi, India; Fundació Institut d’Investigació en Ciències de la Salut Germans Trias i Pujol, Universitat Autònoma de Barcelona, SPAIN

## Abstract

**Introduction:**

There is lack of information on the proportion of new smear—positive pulmonary tuberculosis (PTB) patients treated with a 6-month thrice-weekly regimen under Revised National Tuberculosis Control Programme (RNTCP) who develop recurrent TB after successful treatment outcome.

**Objective:**

To estimate TB recurrence among newly diagnosed PTB patients who have successfully completed treatment and to document endogenous reactivation or re-infection. Risk factors for unfavourable outcomes to treatment and TB recurrence were determined.

**Methodology:**

Adult (aged ≥ 18 yrs) new smear positive PTB patients initiated on treatment under RNTCP were enrolled from sites in Tamil Nadu, Karnataka, Delhi, Maharashtra, Madhya Pradesh and Kerala. Those declared “treatment success” at the end of treatment were followed up with 2 sputum examinations each at 3, 6 and 12 months after treatment completion. MIRU-VNTR genotyping was done to identify endogenous re-activation or exogenous re-infection at TB recurrence. TB recurrence was expressed as rate per 100 person-years (with 95% confidence interval [95%CI]). Regression models were used to identify the risk factors for unfavourable response to treatment and TB recurrence.

**Results:**

Of the1577 new smear positive PTB patients enrolled, 1565 were analysed. The overall cure rate was 77% (1207/1565) and treatment success was 77% (1210 /1565). The cure rate varied from 65% to 86%. There were 158 of 1210 patients who had TB recurrence after treatment success. The pooled TB recurrence estimate was 10.9% [95%CI: 0.2–21.6] and TB recurrence rate per 100 person–years was 12.7 [95% CI: 0.4–25]. TB recurrence per 100 person–years varied from 5.4 to 30.5. Endogenous reactivation was observed in 56 (93%) of 60 patients for whom genotyping was done. Male gender was associated with TB recurrence.

**Conclusion:**

A substantial proportion of new smear positive PTB patients successfully treated with 6 –month thrice-weekly regimen have TB recurrence under program settings.

## Introduction

India accounted for nearly 2.8 million of the estimated 10.4 million global incidence of tuberculosis (TB) in 2016 [1]. The Government of India implemented the Revised National Tuberculosis Control Programme (RNTCP) from 1997, based on the internationally recommended Directly Observed Treatment, Short course (DOTS) strategy. Under RNTCP, newly diagnosed smear-positive pulmonary TB patients were treated with a 6-month thrice-weekly regimen, consisting of an initial intensive phase (IP) of isoniazid (H), rifampicin (R), pyrazinamide (Z) and ethambutol (E) for two months followed by a continuation phase (CP) of H and R for four months (2 H_3_R_3_Z_3_E_3_ / 4H_3_R_3_). Each dose during IP was to be given under direct observation; during CP, the first weekly dose was given under direct observation and the remaining two doses of the week were self-administered [2]. Outcome of treatment is primarily based on follow up sputum microscopy at the end of treatment completion. As per routine surveillance data of RNTCP, treatment success among new microbiologically confirmed pulmonary TB patients registered for treatment with this regimen in the year 2015 was 88%—cured: 84% and treatment completed: 4% [3].

Very few studies were carried out previously, mostly in restricted geographical areas to find out the proportion of such patients who experience TB recurrence and generally involved one time follow up at a variable time point subsequent to treatment completion and thus were limited in study designs [4,5]. There has been lack of information on the rates of recurrence at the national level and the proportion of recurrences attributable to endogenous reactivation / exogenous reinfection. Therefore, a multi-centric study was carried out with the primary objective to estimate the recurrence rate of TB among successfully treated new smear positive pulmonary TB patients within the first year of being declared as cured or treatment completed. It has been demonstrated earlier that the majority of recurrences occur within one year of treatment completion [5]. Secondary objectives included finding out (a) the proportion successfully treated, (b) proportion of recurrent TB cases attributable to endogenous reactivation, (c) risk factors associated with unfavourable TB treatment outcome and recurrence of TB.

## Methodology

### Study setting

**Fig 1:**
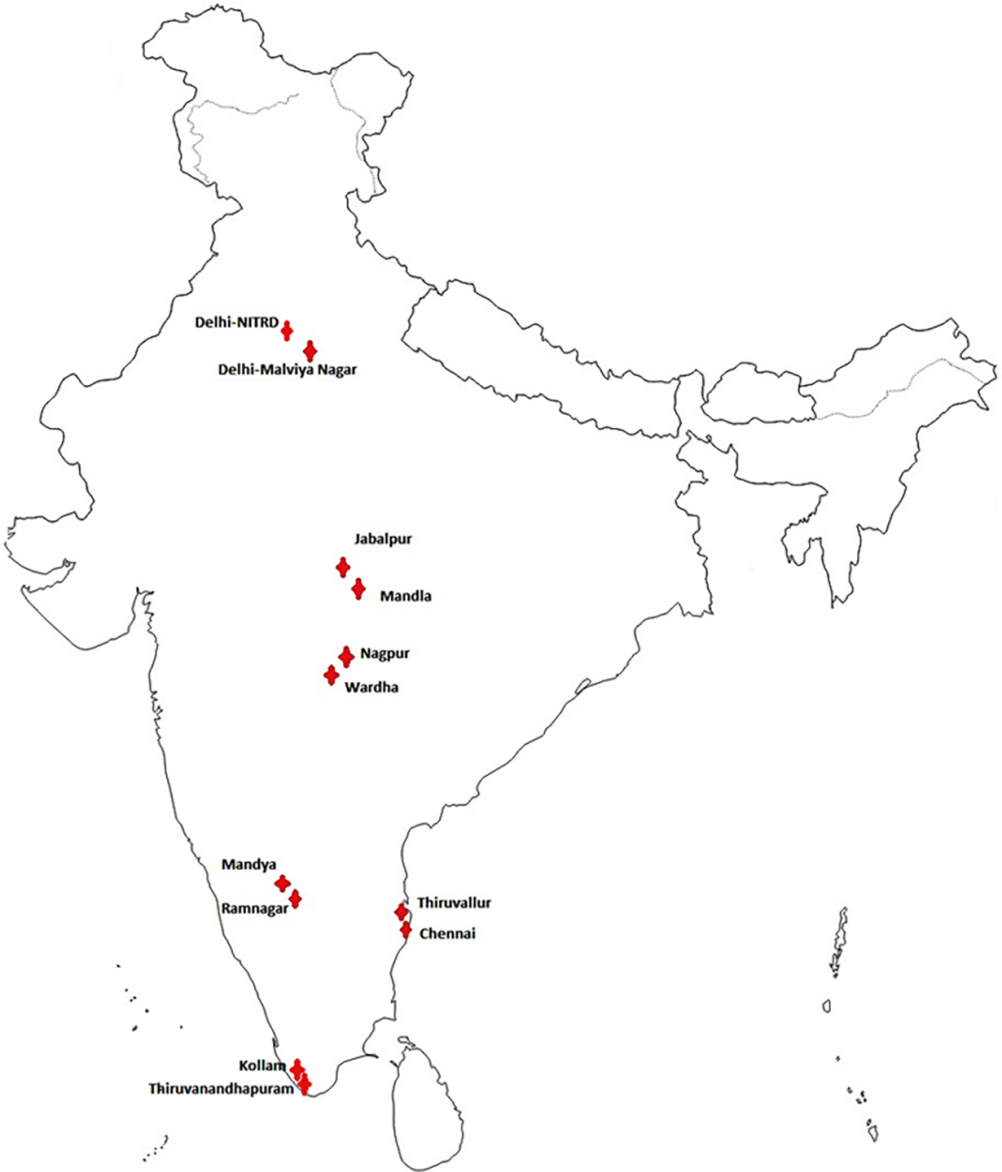
Study sites for the recurrence of TB among the newly diagnosed sputum positive pulmonary TB patients treated under RNTCP in India.

The study was carried out in 12 RNTCP reporting districts by six implementing institutes selected purposively on the basis of relevant research capacity and willingness to participate, with National Institute for Research in Tuberculosis (NIRT), Chennai, as the nodal centre. Three of these institutes were located in the southern part of the country and one each in Northern, western and central. Each implementing Institute carried out the study in two conveniently selected districts (Fig 1). The adult HIV prevalence in 2013–14 in the states of the study sites were as follows: Madhya Pradesh (0.09%), Kerala (0.12%) Delhi (0.22%), Tamil Nadu (0.28%), Maharashtra (0.42%) and Karnataka (0.52%) [6]. The percentage of population below poverty line (2011–12) was 7.1%, 9.9%, 11.3%, 17.4%, 20.9% and 31.6% in Kerala, Delhi, Tamil Nadu, Maharashtra, Karnataka and Madhya Pradesh respectively [7].

### Study population and study design

The study population comprised of new smear positive (NSP) adult (aged ≥ 18 yrs) pulmonary TB patients residing in the selected Tuberculosis Unit (TU) area and registered for treatment with the thrice-weekly intermittent regimen under RNTCP. Of them, those declared successfully treated were followed up prospectively for a period of 12 months post treatment.

### Sampling

The sample size was estimated at 1200 new smear positive pulmonary TB patients registered for treatment; the parameters and values considered for sample size estimation were as under -

Proportion successfully treated = 85%

Proportion of successfully treated patients expected to have TB recurrence = 15%

Absolute precision = 2.5%

Level of significance = 5%

Expected loss to follow-up during the post treatment period = 20%.

The estimated sample size was allocated equally across different implementing institutes, each located in a different state. Thus allocated sample size of 200 each was further distributed equally into 2 districts in the respective state. Within each district, one Tuberculosis units (TU) was selected; the criteria being registration under RNTCP of at least 150 new smear positive pulmonary TB patients in the previous two quarters. In the event of there being no such TU, two TUs were selected. A TU is the sub-district level managerial unit responsible for RNTCP implementation in its geographical jurisdiction inhabited by about 250,000–500,000 population. Within each TU, consecutive patients were recruited into the study till the allocated sample size for the district was achieved.

### Study procedures

The Field Investigators (FI) who were specifically recruited for the study were trained comprehensively and uniformly at NIRT. They visited each Designated Microscopy Centre (DMC) in the respective TUs every week and line listed all diagnosed new sputum smear positive pulmonary TB patients. There are generally 3–5 DMCs in each TU. The FI coordinated with the Senior Treatment Supervisor (STS), Lab Technician (LT), Pharmacist and TB-Health Visitor (TB-HV) for contacting the patient. Those residing outside the study district and <18 years of age were excluded. The FI briefed the patient about the study, its procedures and obtained the patient’s signature in the informed consent form. From those consenting to participate, the FI collected two additional sputum specimens for the purpose of the study prior to initiation of anti-TB treatment (ATT) or within one week of initiating treatment.

Those patients who were not initiated on ATT were excluded from further study procedures. Study procedures included collection of relevant data from RNTCP records, patient interviews, sputum collection and examination by smear and culture, drug susceptibility testing (DST), genotyping of culture isolates, HIV testing and measurement of capillary blood sugar.

#### Data from TB treatment card and patient interview

Initial interview with each patient was conducted by FI—within 14 days of start of treatment. The information that was collected during the interview and from the TB treatment card included age, sex, symptoms with duration, personal habits–smoking / alcohol intake, co-morbid illness–HIV / Diabetes, initial sputum smear grading, height (cm), body weight (kg), date of start of treatment and time interval between onset of symptoms to health seeking and initiation of ATT. The data was recorded in a pre-structured interview schedule.

At the end of the intensive phase of treatment, the information collected from TB treatment card included body weight (kg), sputum smear status, time taken to complete IP, number of doses missed, treatment modification if any, with reason.

At the time of declaration of treatment outcome the following information were collected from the TB treatment card and by second interview with the patient using the “Treatment outcome interview” schedule: body weight (kg), type of treatment outcome with date, number of doses missed, time taken to complete CP, treatment modification if any with reason, symptoms if any, co-morbid illness, personal habits and type of directly observed treatment (DOT)–health facility based / community based. For patients with treatment outcome as ‘loss to follow-up’, attempts were made to interview the patient and also to find out the reasons for interrupting treatment. For “died” patients, the relevant information was obtained by interviewing the relatives of the deceased. Patients not evaluated for treatment outcome due to transfer out were excluded from interview and further follow-up.

For patients declared as successfully treated, a third interview was undertaken at the time of recurrence or at the end of 12 months of post treatment follow-up. The information collected included symptoms if any, whether was ATT taken in the post treatment follow-up period, co-morbid conditions and personal habits.

A minimum of three attempts were made to contact the patient by phone or home visits at each stage of the study, in order to complete data collection.

#### Sputum collection and examination

Two sputum specimens (one spot and one early morning) were collected within 14 days of start of treatment and at the time of treatment outcome. From patients declared as successfully treated, two sputum specimens each were collected at the end of 3^rd^, 6 and 12^th^ months post treatment or at the time of TB recurrence if detected in the intervening periods. The information regarding this was obtained through close liaison with RNTCP staff and periodic telephone calls with the patient.

Each specimen was subjected to sputum smear examination and culture for Acid Fast Bacilli (AFB) at the respective institution’s RNTCP certified mycobacteriology laboratory; three of which were National Reference laboratories (NRL) and the other three were Intermediate Reference laboratories (IRL). Microscopy was performed using Ziehl Neelsen staining method or florescent microscopy depending upon the availability at the respective institute [8]. Sputum culture was undertaken on solid Lowenstein Jensen (LJ) medium as per RNTCP Training Manual for *Mycobacterium tuberculosis* Culture and Drug susceptibility testing (DST) [9]. Results were classified as: No growth–Negative, 1–9 colonies—scanty (actual number of colonies noted), 10 to 100 colonies– 1+, >100 colonies– 2+, confluent growth -3+, Contaminated–Cont, Atypical growth–Non-Tuberculous Mycobacteria (NTM). Identification tests were performed using Niacin Test, Nitrate Reduction Test and MPT64 Rapid lateral flow assay. For growth identified as *M*. *tuberculosis*, Drug Sensitivity Testing (DST) was undertaken at the respective NRL to H, R and E by the Proportion Susceptibility Method (PST) using single concentration of 0.2, 40 and 2 μg/ml respectively [9]. Any strain exhibiting 1% or more of growth on drug slopes in comparison with the corresponding drug free slope was considered resistant. For cultures performed at IRL, the DST was carried out at the respective NRL or at the IRL itself, if approved and certified. DST results were informed to the patient, the Medical Officer (MO) of the Health Centre as well as the district TB officer for appropriate action as per RNTCP guidelines.

#### Genotyping

The positive baseline cultures from all the centres were transported to NIRT to be stored at -80°C for later genotyping in case of TB recurrence. Genotyping was also performed on the growth of culture found positive for *M*. *tuberculosis* at the time of recurrence.

The Mycobacterial culture were retrieved by culturing on to LJ medium and then sub-cultured again on LJ medium to extract the genomic DNA. Genomic DNA was extracted from *M*. *tuberculosis* cultures by standard cetyltrimethyl ammonium bromide (CTAB)–NaCl extraction method.

MIRU-VNTR genotyping was performed by resolution under 2% gel with each locus being amplified separately by simplex PCR [10]. The gel picture thus obtained was used for the sizing. The size of the PCR product was determined by comparison with the position of the size-standard marker. This size was then matched with the MIRU-VNTR Table. The following were verified before assigning the allele number.

- The consistence with the usual allelic range.
- The allele assignation of the H37Rv control is correct.
- The consistency of the results were judged by the incremental spacing between PCR products from different isolates

If the MIRU patterns were identical at the time of diagnosis and at recurrence, the recurrence was attributed to endogenous reactivation (Relapse) and to exogenous re-infection if they differed by 2 or more MIRU-VNTR [10].

#### Blood tests

If the patient was not already tested for HIV, he/she was referred to the Integrated Counseling and Testing Centre (ICTC) at the time of the initial interview and the results were recorded. If the HIV test was negative, it was repeated in the event of the patient having a recurrence of TB.

Capillary blood glucose (CBG) measurement was undertaken using a glucometer after overnight fasting. Blood sugar was recorded from study participants at the time of initial interview and at the time of recurrence of TB. In case of abnormal blood sugar values (fasting capillary glucose level ≥ 110 mg/dl), patients were guided for further evaluation to confirm / rule out diabetes mellitus [11].

#### Study definitions

The definitions used in the study for new TB patient, treatment outcome, TB recurrence, co-morbid condition, personal habits are provided as Supporting information (S1 File)

#### Ethical considerations

The study was approved by the Institutional Ethics Committees of each participating Institute.

Each study participant was informed of the objectives and study procedures using patient information sheet printed in local language as well communicating verbally and written consent was sought. No one was forced to participate in the study. All the patients were treated with standardized regimen at the nearest public health facility as per the RNTCP guidelines.

For patients whose DST results showed resistance to one or more drugs, information was provided to the treating MO for necessary treatment change.

All patients found to be culture positive at the end of treatment were referred to the respective District TB Officer (DTO) /MO-TU under intimation for further necessary treatment. DST results were communicated to the treating MO-TU as soon as available. Similarly, patients found to be recurrent were referred for appropriate treatment along with the smear, culture and DST results.

Attempts were also made to retrieve patients lost to follow-up during treatment and put them on re-treatment regimen depending upon the culture and DST status as per RNTCP guidelines.

An amount of Rs. 200/- (3$) was given to each study participant at the time of each interview, blood or sputum collection as compensation for transport expenses and loss of wages if any.

#### Data analysis

The data was scrutinized for completeness and consistency, and entered using Microsoft Access, 2003/2007. Data was analysed using SPSS version 20.0. Quantitative variables were summarized using mean and standard deviation (SD) or median and inter-quartile range (IQR) as applicable. Proportions were computed for categorical variables. TB recurrence among new smear positive patients who had successful treatment outcome was expressed as rate per 100 person-years with 95% confidence interval (CI). TB recurrence rate per 100 person-years was calculated using Epi-Info 7.0. Since there was no monthly follow-up, the occurrence of event was assumed at the mid time point between the follow-up periods of 3, 6 and 12 months.

The pooled proportion with TB recurrence and the pooled recurrence rate were calculated using the following formula:-

$$P=\frac{\sum_{i=1}^{6}w_{i}p_{i}}{\sum_{i=1}^{6}w_{i}}$$

Where w_i_ is the weight corresponding to i^th^ site and equals 1/Variance of p_i_

p_i_ is proportion /recurrence rate in each site and P is the pooled value_._

Standard error was calculated using formula:-

$$SE=\sqrt{\frac{\sum_{i=1}^{6}w_{i}^{2}{\left(p_{i}-P\right)}^{2}}{\sum_{i=1}^{6}{\left(w_{i}\right)}^{2}}}$$

Relative Risk (RR) and Adjusted RR (ARR) with 95% CI were calculated using the Poisson regression model to identify the potential risk factors for unfavourable response to treatment and for TB recurrence. The variables with p value ≤ 0.10 in the univariate analysis were considered for multiple regression model which was performed by the enter method in which all the variables were entered in a single step; p value <0.05 was considered to be statistically significant.

## Results

**Fig 2:**
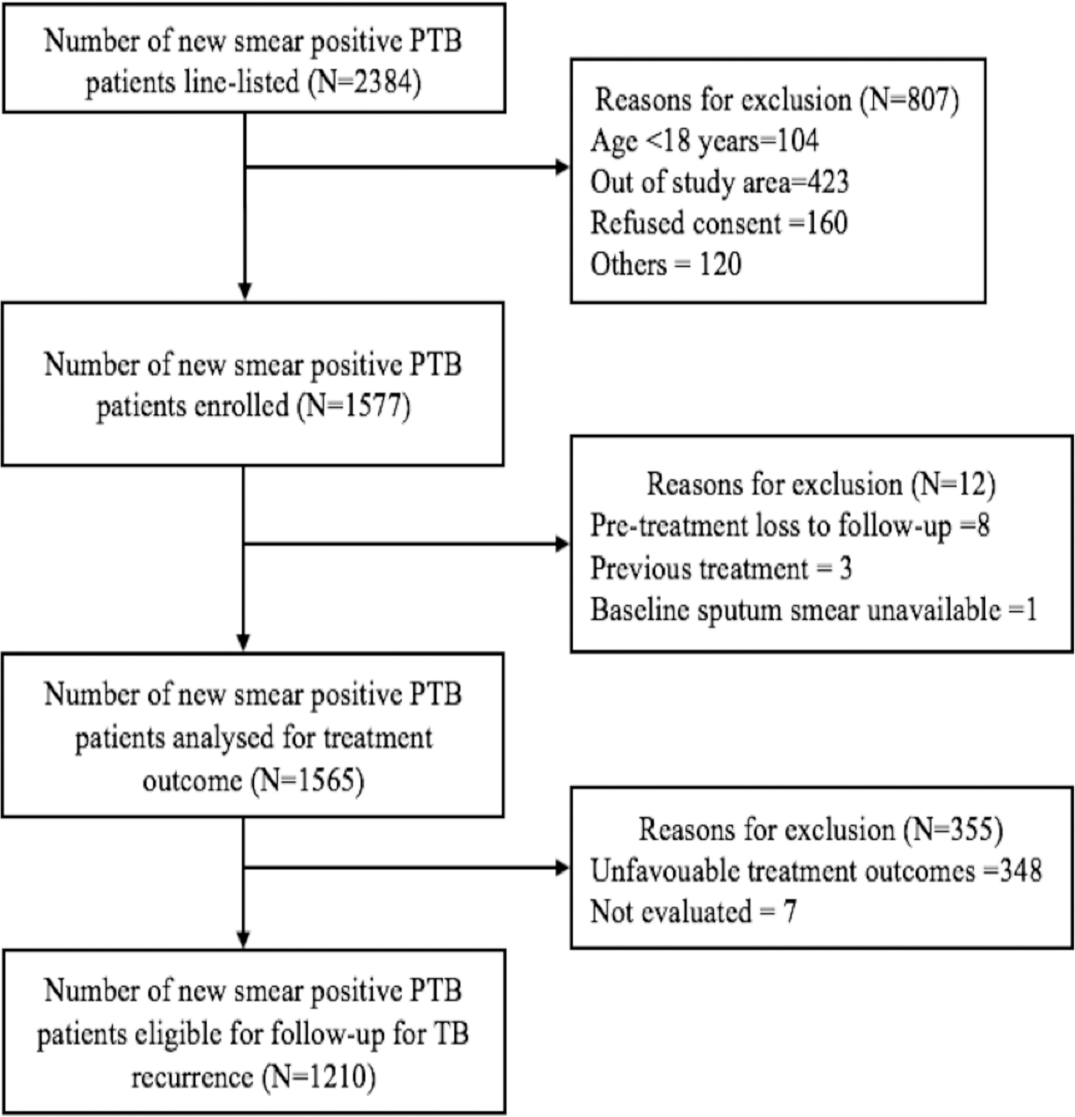
Details on screening, enrolment and follow-up for TB recurrence in new smear positive pulmonary TB patients under RNTCP.

A total of 2384 patients were line listed, of which 1577 were enrolled into the study which varied from 242 to 302 between different sites (Fig 2). The reasons for non-enrolment included age <18 years, residing out of study TU, refused consent and others (sick and hospitalized, domiciliary instability, previous anti-TB treatment). After excluding 12 patients [pre-treatment loss to follow-up (n = 8), previously treated (n = 3), initial smear not available (n = 1)], 1565 patients were available for analysis [Tamil Nadu (n = 249), Karnataka (n = 230), Delhi (n = 302), Maharashtra (n = 246), Kerala (n = 297) and Madhya Pradesh (n = 241)].

### Baseline characteristics

The baseline characteristics of the 1565 patients are shown in Table 1. Majority, 1125 (72%) were males. The mean age ± SD was 41 ± 15 years (range 18 to 90 years) and the mean Body mass index (BMI)± SD was 17 ± 3 Kg/m^2^ (range 10 to 38 Kg/m^2^).

**Table 1:** Baseline characteristics of new smear positive pulmonary TB patients [N = 1565].

Patient characteristics		Total
		n (%)
Sex	Male	1125 (72)
	Female	440 (28)
Age (years)	18–24	279 (18)
	25–34	335 (21)
	35–44	270 (17)
	45–54	307 (20)
	55–64	242 (16)
	≥ 65	132 (8)
Body mass Index (Kg/m^2^)	<16	542 (35)
	16–18.49	511 (33)
	18.5–22.9	403 (26)
	≥23	108 (7)
Smoking	Non-smoker	807 (52)
	Current	86 (5)
	Past	672 (43)
Alcohol use	No	759 (48)
	Current	27 (2)
	Past	779 (50)
HIV status	Reactive	48 (3)
	Non-reactive	1506 (96)
	Not Known	11 (1)
Diabetes mellitus	Yes	293 (19)
	No	1272 (81)

Overall, 884 (56%) were aged 18–44 years. BMI of <18.5 (under-weight) was observed in 1053 (67%) of the total patients. There were 807 (52%) never smokers, 672 (43%) past smokers and 86 (5%) current smokers. The median duration of alcohol intake was 15 years [IQR: 7–25 years]. There were 759 (48%) non/never-alcohol users, 779 (50%) past users and 27 (2%) current users.

Of the 1565 patients, 1506 (96%) were non-reactive for HIV, 48 (3%) reactive and HIV status was unknown for remaining 11. Of the 48 HIV reactive patients, 38 (79%) were put on anti-retroviral treatment.

Two hundred and ninety three (19%)were found to have diabetes. Details of anti-diabetic medications were available for 276 (94%): 183 (65%) were on oral drugs, 54 (19%) on insulin and 39 (14%) on both insulin and oral drugs.

### Baseline sputum smear, culture and drug susceptibility profile

Distribution of study population by sputum smear, culture and DST results is given in Table 2.

**Table 2:** Baseline sputum grading and drug susceptibility profile of new smear positive pulmonary TB patients.

Sputum smear and culture status		Total
		n (%)
Sputum smear maximum		**N = 1565**
grade*	Scanty positive	127 (8)
	1+	578 (37)
	2+	389 (25)
	3+	471 (30)
Sputum culture status		**N = 1565**
	Any one culture *Mtb* positive	1368 (87)
	Culture negative	166 (11)
	Non-Tuberculous mycobacteria	8 (<1)
	Contaminated	18 (1)
	Sample insufficient	5 (<1)
Sputum culture maximum		**N = 1368**
grade	Colonies	60 (4)
	1+	626 (46)
	2+	494 (36)
	3+	188 (14)
Drug susceptibility profile		**N = 1304**
	Susceptible to H, R, E	1173(90)
	Resistant to any drug	131 (10)
	Resistant to H	77
	Resistant to R	6
	Resistant to E	27
	Resistant to HR	3
	Resistant to HE	7
	Resistant to ER	1
	Resistant to HRE	10

*Under RNTCP, as per standard of care, 2 sputum specimens are examined for smear. Grading by Ziehl Neelsen staining as follows: Negative—No AFB in 100 fields, Scanty = 1–9 AFB per 100 fields, 1+ = 10–99 AFB per 100 fields, 2+ = 1–10 AFB per field, 3+ = > 10 AFB per oil immersion field [6]. Grading by Auramine O Fluoresent staining is as follows: Negative—No AFB in 100 fields, 1+ = 1–10 AFB per 100 fields, 2+ = 11–100 AFB per 50 fields, 3+ = > 100 AFB per 20 fields [6].

H: Isoniazid, R: Rifampicin, E: Ethambutol

Of the 1565 patients, 860 (55%) had sputum smear grading of 2+/3+. A total of 1368 (87%) had at least one baseline culture positive for *M*. *tuberculosis*. There were 166 (11%) with negative sputum cultures, 8 (<1%) with cultures positive for *Non-tuberculous mycobacteria*, 18 (1%) with culture contamination and 5 (<1%) with insufficiency of specimens.

Information on drug susceptibility test was available for 1304 (95%) of 1368 culture positive patients. of them, 1173 (90%) were susceptible to H, R and E while the remaining 131 (10%) had resistance to one or more drugs. H mono-resistance was observed in 77 (6%), E mono-resistance in 27 (2%), EH resistance in 6 (<1%), and resistance to R/HR was present in 20 (1.5%).

### Duration from onset of symptoms to anti-TB treatment initiation

The median duration from the onset of symptoms to 1^st^ care seeking was 2 weeks [IQR: 1–6 weeks]. The median duration from 1^st^ care seeking to anti-TB treatment initiation was one week [IQR: 1–5 weeks]. Overall, the median duration from the onset of symptoms to anti-TB treatment initiation was 6 weeks [IQR: 3–12 weeks].

### Sputum smear conversion at the end of intensive phase of treatment

Sputum smear status at the end of IP was available in 1442 (92%) of 1565 patients. Of them 1241 (86%) had converted to smear negative by 2 months of IP and additional 146 (10%) converted by 3 months of IP; the information on month of conversion was missing for one patient; while the remaining 54 had not converted.

### Treatment outcome

Of 1565 patients, 1210 (77%) were successfully treated (cured: 1207, Treatment completed: 3) [Table 3]. Treatment failure was observed in 126 (8%) and 93 (6%) patients died during TB treatment. The period of death from treatment initiation was available for 84 (90%) patients; 44 (52%) died within 60 days of treatment initiation. Ninety-six (6%) patients were lost to treatment follow-up. Reasons for lost to treatment were obtained from 82 (85%) patients; these were alcohol use related in 29 (35%), resolution of symptoms in 20 (24%), side-effects to drugs in 7 (8%), work related in 1 (1%) and others 31 (38%) which included migration/refusal to take medication.

**Table 3:** Treatment outcome of new smear positive pulmonary TB patients [N = 1565].

Treatment outcome	Total
	n (%)
Cured	1207(77)
Treatment completed	3 (<1)
Treatment failure	126 (8)
Died	93 (6)
Lost to treatment follow-up	96 (6)
Switch over to MDR–TB treatment	18 (1)
Treatment modified	15 (1)
Not evaluated	7 (<1)

MDR-TB: Multidrug resistant TB.

There were 18 (1%) of the total who were switched over to MDR-TB treatment. Treatment was modified for 15 (1%) patients: for diabetes (n = 8), clinical indication (n = 6) in whom continuation phase was extended and in one for medical indication (renal failure). There were 7 (<1%) patients who were not evaluated for treatment outcome.

### TB recurrence

Of 1210 successfully treated patients, 950 (79%) had recurrence free survival at 12 months follow-up, 158 (13%) had recurrence at variant time points during the period of follow-up, 73 (6%) were lost to follow-up by 12 months and 29 (2%) had died [Table 4]. The weighted proportion of patients who had TB recurrence was 10.9% [95%CI: 0.2–21.6] and TB recurrence rate per 100 person–years was 12.7 [95% CI: 0.4–25].

**Table 4:** Status at 12 months after Treatment success in new smear positive pulmonary TB patients [N = 1210].

Status of patients	Total
	n (%)
No TB	950 (79)
TB recurrence	158 (13)
Lost to follow-up	73 (6)
Died	29 (2)
Weighted proportion of patients with TB recurrence	10.9% [95%CI: 0.2–21.6]
Weighted TB recurrence rate per 100 person years	12.7 [95% CI: 0.4–25]

CI: Confidence Interval

Pulmonary TB constituted 155 (98%) of the 158 TB recurrences, and 3 had TB lymphadenitis. Of PTB patients, 150 were bacteriologically positive [Table 5].

**Table 5:** Details of TB recurrence in new smear positive pulmonary TB patients [N = 158].

TB recurrence		Total
		n (%)
**Type of TB**		
	Bacteriologically confirmed Pulmonary TB	150(95)
	Clinically diagnosed Pulmonary TB	5 (3)
	Clinically diagnosed Extra-pulmonary TB	**3 (2)**
**Month of TB recurrence post-treatment**		
	0–3 months	87 (55)
	4–6 months	35 (22)
	7–12 months	36 (23)
**Genotyping of TB recurrence**		
	Culture positive at baseline and TB recurrence	123
	Endogenous reactivation	56
	Exogenous re-infection	4
	DNA could not be extracted	63

A total of 87 (55%) patients had TB recurrence within 3 months post-treatment and a total of 122 (77%) occurred within 6 months.

Of the 150 patients who had bacteriologically confirmed TB recurrence, 123 had available baseline and recurrent sputum cultures positive for *M*.*tb* and were eligible for genotyping. Genotyping details were available for 60 (49%) of 123 patients while the DNA could not be extracted in the remaining 63 patients. Endogenous reactivation was observed in 56 (93%) patients. Two HIV positive patients with recurrence had endogenous reactivation. Genotyping could not be done in 63 patients since DNA could not be extracted due to inadequate growth or contamination.

### Risk factors for unfavourable outcome of anti-TB treatment

The factors analyzed for unfavourable response at the end of treatment included sex, age-group, BMI, initial sputum smear grade, culture grade, drug susceptibility profile, diabetes and anti-diabetic medications, HIV and ART status, smoking, alcohol, duration of intensive phase of treatment, place of treatment, duration from symptoms to treatment initiation and missed doses in intensive phase (Table 6). None of the factors analysed were found to be associated with unfavourable response to treatment.

**Table 6:** Risk factors for unfavourable response at the end of treatment in new smear positive pulmonary TB patients [N = 1543].

Characteristics		No. evaluated for treatment outcome N = 1543*	Unfavourable response				RR [95% CI]	p value	ARR[95% CI]	p value
			Yes N = 333		No N = 1210					
		N	n	%	N	%				
**Sex**	Male	1111	270	24	841	76	1.08[0.98–1.20]	0.12		
	Female	432	63	15	369	85	Ref			
**Age-group (years)**	≥65	130	26	20	104	80	1.01[0.84–1.23]	0.86		
	55–64	238	59	25	179	75	1.06[0.90–1.24]	0.48		
	45–54	304	67	22	237	78	1.03[0.89–1.20]	0.66		
	35–44	268	61	23	207	77	1.04[0.89–1.21]	0.61		
	25–34	325	70	22	255	78	1.03[0.89–1.19]	0.69		
	18–24	278	50	18	228	82	Ref			
**Body Mass Index**	≥23	104	6	6	98	94	0.89[0.72–1.09]	0.28		
**(Kg/m**^**2**^**)**	16–18.4	506	117	23	389	77	1.04[0.92–1.17]	0.54		
	< 16	535	136	25	399	75	1.06[0.94–1.19]	0.35		
	18.5–22.9	398	74	19	324	81	Ref			
**Baseline sputum**	2+ / 3+	850	193	23	657	77	1.02[0.93–1.12]	0.66		
**smear grade**	Scanty/ 1+	693	140	20	553	80	Ref			
**Baseline sputum**	2+ / 3+	671	142	21	529	79	0.98[0.89–1.08]	0.64		
**culture grade**	Cols/ 1+	676	162	24	514	76	Ref			
**Baseline drug**	Resistant	131	50	38	81	62	1.15[0.98–1.34]	0.08	1.14[0.96–1.35]	0.14
**susceptibility test**	Sensitive	1152	232	20	920	80	Ref		Ref	
**Smoker**	Current	87	31	36	56	64	1.16[0.96–1.41]	0.12		
	Past	664	170	26	494	74	1.08[0.98–1.18]	0.12		
	Non-smoker	792	132	17	660	83	Ref			
**Alcohol use**	Current	27	5	18	22	82	1.02[0.72–1.46]	0.90	1.00[0.68–1.47]	1.00
	Past	767	209	27	558	73	1.09[1.00–1.20]	0.04	1.08[0.97–1.20]	0.17
	No	749	119	16	630	84	Ref		Ref	
**Diabetes mellitus** and anti-diabetic medications	Not on anti-diabetic treatment	114	16	14	98	86	0.92[0.77–1.11]	0.39		
	On anti-diabetic treatment	169	22	13	147	87	0.92[0.79–1.06]	0.25		
	No-diabetes	1260	295	23	965	77	Ref			
**HIV status**	Reactive, not on ART	11	9	82	2	18	1.51[0.97–2.34]	0.07	1.44[0.68–3.03]	0.34
	Reactive, on ART	38	13	34	25	66	1.11[0.84–1.47]	0.45	1.04[0.73–1.46]	0.84
	Non-reactive	1491	308	21	1183	79	Ref		Ref	
**Duration of**	3 months	193	47	24	146	76	1.09[0.95–1.24]	0.24		
**Intensive Phase**	2 months	1237	180	15	1057	85	Ref			
**Type of DOT**	Community based	532	108	20	424	80	1.03[0.94–1.34]	0.53		
	Health centre based	912	151	17	761	83	Ref			
**Duration from**	>12	292	75	26	217	74	1.03[0.90–1.18]	0.63		
**symptoms to**	9to 12	208	40	19	168	81	0.98[0.84–1.14]	0.79		
**treatment**	6to 9	244	53	22	191	78	1.00[0.87–1.15]	0.99		
**initiation (weeks)**	3to 6	373	73	20	300	80	0.98[0.87–1.12]	0.79		
	≤3	426	92	22	334	78	Ref			
**Missed doses in**	>12	23	11	48	12	52	1.31[0.93–1.84]	0.12	1.29[0.91–1.81]	0.15
**Intensive phase**	7 to 12	43	19	44	24	56	1.28[0.99–1.65]	0.06	1.21[0.91–1.59]	0.19
**of treatment**	1 to 6	175	43	25	132	75	1.10[0.96–1.27]	0.18	1.10[0.95–1.29]	0.20
	No	1190	154	13	1036	87	Ref		Ref	

*excluding not evaluated and treatment modified; RR—Relative risk, ARR—Adjusted relative risk, CI: Confidence Interval

### Risk factors for TB recurrence

The factors analysed for TB recurrence included sex, age-group, BMI, baseline sputum smear grade, culture grade, drug susceptibility profile, diabetes and anti-diabetic medications, HIV and ART status, duration of intensive phase of treatment, type of DOT, smoking and alcohol use during treatment and / or follow-up, respiratory symptoms at the end of treatment, duration of anti-TB treatment, weight gain during treatment and missed doses in intensive phase (Table 7). Of all the factors analysed in 1108 patients with treatment success, being male {ARR: 2.4 [95%CI:1.3–4.6] (p = 0.006)} was associated with TB recurrence.

**Table 7:** Risk factors for TB recurrence in successfully treated new smear positive pulmonary TB patients [N = 1108].

Characteristics		No. successfully treated N = 1108	TB recurrence				RR [95% CI]	p value	ARR[95% CI]	p value
			Yes N = 158		No N = 950					
		N	n	%	N	%				
**Sex**	Male	769	132	17	637	83	2.23[1.47–3.40]	<0.001	2.43[1.29–4.58]	0.006
	Female	339	26	8	313	92	Ref		Ref	
**Age-group (years)**	≥65	90	9	10	81	90	0.75[0.35–1.61]	0.47		
	55–64	163	17	10	146	90	0.79[0.43–1.45]	0.45		
	45–54	227	34	15	193	85	1.13[0.68–1.88]	0.61		
	35–44	190	33	17	157	83	1.31[0.79–2.19]	0.28		
	25–34	233	38	16	195	84	1.23[0.75–2.02]	0.39		
	18–24	205	27	13	178	87	Ref			
**Body Mass Index**	≥23	88	9	10	79	90	0.99[0.47–2.07]	0.97	0.77[0.26–2.27]	0.64
	16–18.4	360	53	15	307	85	1.42[0.91–2.21]	0.12	0.99[0.59–1.67]	0.99
	< 16	361	65	18	296	82	1.73[1.13–2.66]	0.01	1.22[0.73–2.05]	0.44
	18.5–22.9	299	31	10	268	90	Ref		Ref	
**Baseline sputum**	2+ / 3+	607	94	15	513	85	1.21[0.88–1.66]	0.23		
**smear grade**	Scanty/ 1+	501	64	13	437	87	Ref			
**Baseline sputum**	2+ / 3+	487	77	16	410	84	1.05[0.76–1.46]	0.73		
**culture grade**	Cols/ 1+	468	70	15	398	85	Ref			
**Baseline drug**	Resistant	71	10	14	61	86	0.92[0.48–1.76]	0.81		
**Susceptibility test**	Sensitive	848	129	15	719	85	Ref			
** Diabetes mellitus** and anti-diabetic medications	Diabetic not on anti-diabetic treatment	85	6	7	79	93	0.45[0.20–1.02]	0.06	0.34[0.12–1.12]	0.07
	Diabetic on anti-diabetic treatment	138	14	10	124	90	0.65[0.37–1.12]	0.12	1.02[0.48–2.20]	0.94
	Non-diabetic	885	138	16	747	84	Ref		Ref	
**HIV status**	Reactive, not on ART	2	0	0	2		Indeterminate			
	Reactive, on ART	19	4	21	15	79	1.49[0.55–4.01]	0.43		
	Non-reactive	1087	154	14	933	86	Ref			
**Duration of**	3 months	128	14	11	114	89	0.73[0.42–1.27]	0.28		
**Intensive Phase**	2 months	973	144	15	829	85	Ref			
** Type of DOT**	Community based	402	57	14	345	86	0.98[0.70–1.35]	0.89		
	Health center based	690	100	14	590	86	Ref			
**Smoking during**	Yes	429	78	18	351	82	1.53[1.12–2.10]	0.007	1.13[0.70–1.84]	0.61
**treatment and or**	No	651	77	12	574	88	Ref		Ref	
**follow-up**										
**Taking alcohol**	Yes	428	76	18	352	82	1.46[1.07–2.00]	0.017	1.06[0.66–1.71]	0.81
**during treatment**	No	652	79	12	573	88	Ref		Ref	
**and or follow-up**										
**Respiratory**	No	614	101	16	513	84	2.33[0.95–5.7]	0.06	2.43[0.97–6.07]	0.06
**symptom at end**	Any	71	5	7	66	93	Ref		Ref	
**of treatment**	symptom									
**Duration of**	>28	294	42	14	252	86	1.85[0.25–13.49]	0.54		
**anti-TB treatment**	>24 to 28	796	115	14	681	86	1.87[0.26–13.44]	0.53		
**[weeks]**	24	13	1	8	12	92	Ref			
**Weight gain (kg)**	>14	34	3	9	31	91	0.61[0.19–1.94]	0.40		
**from baseline to**	10.01–14.0	64	7	11	57	89	0.76[0.35–1.65]	0.48		
**end of treatment**	6.01–10.0	202	28	14	174	86	0.96[0.62–1.49]	0.85		
	≤ 2	187	31	17	156	83	1.15[0.75–1.75]	0.53		
	No	141	21	15	120	85	1.02[0.63–1.68]	0.90		
	2.01 to 6.0	470	68	14	402	86	Ref			
**Missed doses in**	>12	11	4	36	7	64	2.72[1.00–7.36]	0.05	1.22[0.73–2.05]	0.44
**Intensive phase**	7 to 12	20	4	20	16	80	1.50[0.55–4.05]	0.43	0.99[0.59–1.67]	0.99
**of treatment**	1 to 6	121	23	19	98	81	1.42[0.91–2.22]	0.12	0.77[0.26–2.27]	0.64
	No	950	127	13	823	87	Ref		Ref	

RR—Relative risk, ARR—Adjusted relative risk, CI: Confidence Interval

A higher proportion of HIV reactive patients had recurrence compared to HIV non-reactive patients; however, this difference was not significant due to small numbers of HIV reactive patients.

## Discussion

This is the first study which has attempted to determine the TB recurrence rates of 6 month thrice-weekly regimen under program settings in India, among new smear positive patients distributed across different geographical locations. We observed a TB recurrence rate of 12.7 per 100 person-years within first year after being declared as successfully treated. In terms of proportions, 10.9% experienced recurrence, which was similar to earlier studies carried out in different geographical locations in the country at different time points. A systematic review reported 10% recurrence after successful treatment among patients treated under DOTS regimen in India [12]. In an earlier study in Tamil Nadu state, South India, 12% of successfully treated TB patients were observed to have recurrence when sputum specimen were collected at 6, 12 and 18 months post-treatment and examined by culture [5]. Studies in Gujarat and Bangalore reported the proportions of recurrence at 10.8% and 11.4% respectively when patients were subjected to one time interview at 2 years and 2.5 years after treatment success; these studies were limited in their approach including recall bias [4, 13]. In our study, 77% of observed recurrences occurred within 6 months. In the study in Tamil Nadu as above, 77% of all the recurrences observed over 18 months occurred within 6 months and 92% within 12 months [5].

Being male was more likely to be associated with TB recurrence in our study. Our finding was contrary to an earlier systematic review from India which reported that sex was not a risk factor for TB relapse [12]. Risk factors for TB recurrence reported in earlier studies included diabetes, HIV, smoking, alcoholism, initial drug resistance and drug irregularity [12,14,15]. However, no such association was observed in the present study which may be due to the limitation that the study especially the sample size was not specifically designed for this purpose. Since pulmonary TB is well known to be much more common in males compared to females, it would be reasonable to presume that TB recurrence can also have such such proclivity. We would however like to re-iterate that the study was not specifically designed to identify risk factors. The observation that males were more likely to have TB recurrence needs to be explored in future studies.

TB recurrence was mainly due to endogenous reactivation as suggested by genotyping findings in our study. This reflects poorly on the efficacy of the regimen under program settings in its ability to offer a relapse free cure. Previous molecular study from India has documented that most (88%) of TB recurrences in HIV-infected patients were due to exogenous re-infection while most (91%) of recurrent TB cases in HIV uninfected were due to endogenous reactivation [16]. Similarly, in our study, most (93%) of the recurrent TB cases in HIV uninfected patients were due to endogenous reactivation.

The cure rate in our study was 77%. This is lower compared to the RNTCP target of 90% [17]. The possible reason could be due to the definition of cure in our study which included smear and culture negativity while RNTCP considers only sputum smear negativity. Studies done in new smear positive pulmonary TB patients in different parts of India have reported cure rate of 77% in Mangalore (n = 212), 83.8% in Himachal Pradesh (n = 130), 74% in Tiruvallur District, Tamil Nadu (n = 295) [18–20]. The 8% failure rate observed in our study is twice than the expected RNTCP target of 4% while the overall proportion of deaths during treatment and lost to treatment follow-up were marginally higher at 6% each than the expected target of 5% [17]. Previous studies have documented male, age, alcoholism, smoking, multi-drug resistant TB, weight <35 kg and missed doses to be associated with lost to treatment follow-up, failure or death [4, 20–23]. However, in the present study, none of the factors was found to be associated with treatment outcome which may again be due to the study not having been designed for the purpose.

Sputum conversion of 96% at the end of intensive phase of treatment is according to the RNTCP target of >90% [17]. In addition, we observed that 89% of those had converted by 2 months. A study in Guwahati documented 84% smear conversion at the end of 2 months in 100 new smear positive PTB patients [24]. On the contrary, a retrospective study from Tumkur District in India documented 66.7% sputum smear conversion at the end of 2 months of treatment in 268 new smear positive PTB patients [25]. We did not attempt to collect sputum as part of study procedures at the end of intensive phase of treatment and documented the sputum smear status from TB treatment cards.

We observed a median delay of 6 weeks from onset of symptoms to treatment initiation. A systematic review on delay in diagnosis and treatment of pulmonary TB patients in India reported a median delay of 57.5 days from onset of symptoms to treatment initiation [26]. The median delay from onset of symptoms to 1^st^ care seeking was 19.4 days in the systematic review compared to 2 weeks observed in our study [26]. The variation across sites for the median delay from onset of symptoms to treatment initiation in our study which ranged from 3 weeks to 12 weeks emphasizes the importance of identification of patient and provider related reasons and developing novel strategies to reduce the delay.

The study has few inherent limitations. We did not collect data on the radiological extent of disease. The details of smoking and alcohol were self reported. We were unable to do genotyping on all positive recurrent TB cultures due to insufficient growth.

Our findings suggest that a significant proportion of new smear positive pulmonary TB patients successfully treated with 6-month thrice-weekly regimen have TB recurrence under program settings. The RNTCP has now revised the regimen and the current recommendation is a daily 6-month regimen with Fixed dose combination (FDC) drugs.

## Supporting information

S1 File Study definitions. (DOCX)
