# Supplementary material for: Recurrence of tuberculosis among newly diagnosed sputum positive pulmonary tuberculosis patients treated under the Revised National Tuberculosis Control Programme, India: A multi-centric prospective study
Source: PLoS One. 2018 Jul 6;13(7):e0200150. doi: 10.1371/journal.pone.0200150 (PMC6034867; doi:10.1371/journal.pone.0200150)
Supplement: S1 File — (DOCX) [file pone.0200150.s001.docx]

# **Supporting Information –S1**

# **Study definitions**

**New TB case:** A TB patient who has never received ATT or has received ATT for less than one month

**Type of Treatment outcome**

1. **Successfully treated:** includes the following two categories:
2. **Cured**: A microbiologically confirmed case whose sputum at the end of treatment upon smear examination in DMC was negative and had a negative result of additional sputum specimen collected at the end of treatment on culture performed in NRL/IRL.
3. **Treatment completed**: A patient who has completed treatment and for whom smear or culture examination at the end of treatment has not been done [1].
4. **Unfavourable treatment outcome:** includes the following four categories:
5. **Treatment Failure:** A patient whose Sputum specimen collected at the end of treatment was either positive by smear at DMC and / or positive by culture in the respective IRL/NRL.
6. **Died:** A patient who has died during the course of anti-TB treatment.
7. **Lost to treatment follow-up:** A patient whose treatment was interrupted for ≥1 month [12].
8. **Switched over to MDR-TB treatment:** A patient whose treatment was changed to regimen for multi-drug resistant (MDR) TB after drug susceptibility test result showed drug Resistance to Rifampicin with or without resistance to INH.
9. **Treatment modified:** A patient for whom TB treatment regimen was modified for reasons other than MDR or CP was extended due to clinical reasons.
10. **Not evaluated:** A patient who has been transferred to another TU / district / state and whose treatment outcome is not available.

**Status of the patient at 12 months follow-up**

1. **Recurrence of TB:** A successfully treated patient who has a positive smear or culture at any time during the post treatment follow-up period. In addition, a patient would be considered to have recurrent TB if anti-TB treatment was restarted for clinical/radiographic deterioration (suggestive of TB) or extra-pulmonary TB or if he/she had been re-treated outside RNTCP.
2. **Recurrence free survival:** A successfully treated patient alive at the end of 12 months follow-up and not found to have recurrence.
3. **Died:** A successfully treated patient who died for any reason during the post treatment follow-up period.
4. **Lost to follow-up:** A successfully treated patient whose status at the end of 12 months post-treatment could not be assessed.

**Diabetic:** A patient who was a known or newly diagnosed diabetic.

**Past smoker:** A person who smoked in the past and discontinued by the time of the interview.

**Current smoker:** A person who smoked around the time of the interview either daily or occasionally.

**Never-smoker:** A person who is not a current or past smoker.

**Past alcohol user:** A person who took alcohol in the past and discontinued by the time of the interview.

**Current alcohol user:** A person who admits to take alcohol around the time of the interview either daily or occasionally.

**Never-alcohol user:** A person who has never consumed alcohol.

**Body mass Index (BMI):** BMI was calculated by dividing observed weight in kg by height in meters squared (kg/m^2^). Patients were classified as underweight (<18.5), normal (18.5 -22.9) and overweight (>23) [2].

**Multi-drug resistant TB** (**MDR-TB):** Resistance to both Isoniazid and Rifampicin.

**Missed Dose:** Not consumed drugs on due date.

**Type of Directly observed treatment (DOT)** was classified as *health centre* based if treatment was given in General Hospital, Primary Health Centre or Sub-centre while the others were classified as *community* based (eg. patients’s residence/work spot, provider’s residence/work spot)

# **References**

1. Central TB Division. Revised definitions for RNTCP Modular training. www.rntcp.nic.in.
2. WHO/IASO/IOTF. The Asia-Pacific prospective. Redefining obesity and its treatment. Sydney: Health Communications: 2000.
